# Ethical issues evolving from patients’ perspectives on compulsory screening for syphilis and voluntary screening for cervical cancer in Kenya

**DOI:** 10.1186/1472-6939-15-27

**Published:** 2014-03-28

**Authors:** Dickens S Omondi Aduda, Nhlanhla Mkhize

**Affiliations:** 1 School of Public Health, Maseno University, Maseno, Kenya; 2 School of Psychology, University of KwaZulu Natal, Pietermaritzburg, South Africa

**Keywords:** Ethics, Public health, Compulsory screening, Voluntary screening, Participants’ perspectives, Syphilis, Cervical cancer

## Abstract

**Background:**

Public health aims to provide universal safety and progressive opportunities to populations to realise their highest level of health through prevention of disease, its progression or transmission. Screening asymptomatic individuals to detect early unapparent conditions is an important public health intervention strategy. It may be designed to be compulsory or voluntary depending on the epidemiological characteristics of the disease. Integrated screening, including for both syphilis and cancer of the cervix, is a core component of the national reproductive health program in Kenya. Screening for syphilis is compulsory while it is voluntary for cervical cancer. Participants’ perspectives of either form of screening approach provide the necessary contextual information that clarifies mundane community concerns.

**Methods:**

Focus group discussions with female clients screened for syphilis and cancer of the cervix were conducted to elicit their perspectives of compulsory and voluntary screening. The discussions were audiotaped, transcribed and thematic content analysis performed manually to explore emerging ethics issues.

**Results:**

The results indicate that real ethical challenges exist in either of the approaches. Also, participants were more concerned about the benefits of the procedure and whether their dignity is respected than the compulsoriness of screening *per se*. The implication is for the policy makers to clarify in the guidelines how to manage ethical challenges, while at the operational level, providers need to be judicious to minimize potential harms participants and families when screening for disease in women.

**Conclusions:**

The context for mounting screening as a public health intervention and attendant ethical issues may be more complex than hitherto perceived. Interpreting emerging ethics issues in screening requires more nuanced considerations of individuals’ contextual experiences since these may be contradictory to the policy position. In considering mounting screening for Syphilis and cervical cancer as a public heal intervention, the community interests and perspectives should be inculcated into the program. Population lack of information on procedures may influence adversely the demand for screening services by the individuals at risk or the community as a collective agent.

## Background

Public health aims to provide universal safety and progressive opportunities to populations to realise their highest level of health through prevention of disease, its progression or transmission [1]. Screening as a public health strategy entails early detection of disease or its precursors in asymptomatic populations deemed to be at risk [2-5]. Its key objectives and rationale include detecting diseases early when treatment is more cost-effective; identifying disease predisposing factors and appropriate management of identified risk factors [2,3]. Depending on the epidemiological characteristics of the disease, defined by its occurrence and resource availability for care and management, screening may be mounted as voluntary or mandatory national program with a long-term aim of reducing morbidity and mortality [2,6-8].

The process of screening to detect early different diseases inherently is fraught with diverse ethical challenges [6,9]. However, analysis of these challenges may be taxing to decision-makers and service providers since ethics constructs for considering public health practice are varied [10,11]. Ethical analysis is needed to clarify the contextual level of public health necessity as well as evaluate the rationale for, prioritize and justify activities designed to accomplish the stated public health objectives [12]. Policies on screening for syphilis and cancer of the cervix in Kenya vary given their epidemiological as well as medical characteristics [13-16]. They were considered simultaneously in the current study to help clarify ethical issues in screening for them based on the perceptions of women undergoing the process.

### Epidemiological and policy context for screening in Kenya

The prevalence of antenatal syphilis in Kenya is estimated at 3.8% and more than half of these develop unfavorable obstetric outcomes, such as maternal deaths, prolonged morbidity and congenital fetal syphilis. Untreated syphilis pose high threats to the general population because of its potential for an outbreak [17,18]. Screening for maternal syphilis early in the first trimester is a mandatory exercise aimed at minimizing the adverse outcomes to the mother and the unborn child as well as to control disease spread or potential epidemics in the general population. This is plausible since syphilis is an infectious but preventable and easily treatable disease, although on the other hand, this may be perceived as a way of compulsion and control. While sufficient service coverage is necessary, apparently few antenatal mothers access these services as stipulated given that majority of women in Kenya initiate antenatal clinic visits late (at a mean gestation of 5.9 months). Even then, less than one third of receive relevant information and fewer ever obtain blood tests [19].

Cervical cancer on the other hand, is the leading cause of reproductive tract cancers in Kenya. It is associated with high morbidity and mortality burden among women at risk yet it is largely preventable and can be effectively treated if detected early [7]. Consequently, its prevention through health promotion and education activities, early detection through screening and effective management of cases has been prioritized in the National Reproductive Health Strategy. The national operational guidelines are available to enhance procedure administration. It is stipulated that providers should take informed consent and document adequate reproductive health history during screening. Additionally, after the procedures results as well as plans for return visits should be discussed with the client. These steps help to improve both clients’ pre- and post-screening psycho-cognitive status [20]. However, it is still unclear how patients experience screening procedures/services vis-à-vis their perceptions/experiences of cervical cancer.

As the Kenya national public health policy goals evolve, elimination of disease [21]; community participation [22]; promotion of effective, accessible, acceptable and affordable quality health services [23] and enhanced regulatory capacity of Ministry of Health (MoH) have been critical [24]. The recent national health guidelines [7,20,22-26] have progressively adopted a life-cycle, rights-based essential health care, presuming the right to ‘the highest attainable standard of health’ for everyone. The core values of a rights-based approach include fairness, respect, equality, dignity and autonomy [27] as well as accountability and community participation [28].

Under the national reproductive health program [29] whose aim is to improve maternal health; reduce neonatal/child mortality and morbidity; reduce the spread of HIV/AIDS and promote empowerment of women, integrated screening, including for both syphilis and cancer of the cervix, has been incorporated as part of the essential health package [20]. These services are largely integrated within the existing outpatient departments [29], maternity units and comprehensive care clinics at all health service tiers [7,30]. Specifically for syphilis, the Public Health Act in Chapters 45 (a) – (c), 48 and 51 provides for mandatory surveillance to identify localities of high disease prevalence and undertaking of appropriate medical and public health interventions. However, the Act also provides that services must be provided in the context of informed consent, privacy, confidentiality, information, education and appropriate communication. Further, a client or her partner(s) must be notified once the disease is diagnosed and provided with written instructions about the condition, appropriate education and counseling while, ensuring strict privacy and confidentiality of their medical records [7,31].

There are challenges in studying or implementing programs for Syphilis and cervical cancer given they are associated with sex and reproduction. Social or psychological status may be harmed by positive results due to loss of personal and social esteem, stigma, anxiety and fetal loss, among others. On the other hand, curveillance and disease notification within the context of screening are necessary in identifying proportionate distributions of disease burden in populations as well as the level of prevention and care resources required [10,12]. The fundamental policy and operational challenge remains how to achieve a balance in effective population coverage while minimizing potential harms to individuals or communities [32]. These potential harms have not been sufficiently explored in Kenya. This study sought to explore women’s perception of mandatory screening for syphilis and voluntary screening of cervical cancer in Nyanza, Kenya with an aim to identify evolving ethical issues. The results highlight local interpretative notions useful for progressive planning and implementation decisions [33,34].

## Methods

### Study design and survey instruments

This qualitative study was conducted in 2009 among clients attending cervical cancer and antenatal clinics in Nyanza Provincial hospital; Kisumu District hospital and Lumumba Municipal health center, all within Kisumu City. The purpose was to explore ethics issues related to both compulsory and voluntary screening from clients’ perspectives. Focus Group Discussions (FGDs) were conducted using interview guides adapted from the ‘Alliance for Cervical Cancer Prevention - Reproductive Health Reports Number 4, July 2001(ACCP) [13] which contains standard questions for evaluating cervical cancer screening in the developing world.

Discussion points, among other issues, included the participants’ knowledge of the target disease; concerns about developing the disease; perceptions of their own personal risk of Cancer of the cervix or Syphilis and acceptable options for preventions respectively; their motivations to attend screening clinics; disease expectancies (specifically related to their beliefs about the source and consequences of the disease); communicating risk and test results to family members; perceptions, feelings, concerns and experiences during screening either for Syphilis or Cancer of the cervix; repeat tests, privacy, confidentiality and access to services. The FGDs were tape-recorded and supplemented with back-up observer notes (which captured in summary important relevant issues, such as emotional contents, demeanor and overall ‘feelings’ of the focus group).

### Eligibility and recruitment procedure

Participants were recruited over three weeks through a two stage process (to minimize potential for coercion). First, the service providers sensitized their clients about the study, giving only basic information about the study. This was to give all clients undergoing the tests an opportunity to choose to participate, while avoiding potentially biased approach by study staff. Subsequently, those who were willing to participate in a discussion group were referred (using a study referral note) to the research assistants sitting in a different room. It was considered that those who presented to the study staff were truly willing to participate. However, the study staff conducted consenting to every client individually by providing in-depth information about the study and answering questions arising.

Those willing to participate in the focus group provided written consent. Only females aged at least 18 years and had been tested for Syphilis or undergone cervical cancer screening that day were consented and enrolled. During consenting, everyone was assured of confidentiality and privacy; informed that their voices will be audio-tapped and that they should not use their names during the discussions. Each was assigned a unique number to use during the focus group discussions. Five to eight participants were enrolled sequentially into each focus group. Recruitment was stopped when no new or relevant data seemed to emerge from further interviews. Sixty four women participated in these focus group discussions. Two assistants, a moderator and note taker conducted the group discussions.

Each session lasted about 45 – 60 minutes. Kiswahili (one of the national languages) was the primary language of communication, since majority understood it well. All focus groups were conducted within the facility on the same day participants were recruited. Building consensus on sessions’ ground rules, and conducting the sessions in a secluded place by female research assistants helped particularly to reassure participants. A snack was provided to participants during the session. No other compensation was provided. After each session, the research assistants alerted the service provider who then promptly attended to the client in case they were not yet through with any of the clinic procedure such as getting feedback, to avoid further inconvenience.

All regulatory and administrative approvals were granted by the Ethics Review Committee (ERC) at University of KwaZulu Natal (school of psychology) and Kenya’s National Council for Science and Technology (NCST). Also, relevant authorizing officers from the Ministries of Health headquarters, Provincial administration at the district level, public health department at the Kisumu City council, Nyanza Provincial and Kisumu district hospitals endorsed copies of the approval letters and filed them.

### Analysis

The moderator for each group transcribed and translated the audio-recordings into English. The first author [DO] reviewed every transcription to confirm accuracy and content validity based on the original audio-recordings. During the translation, we tried to keep the phrases and words as in the spoken language, to capture the context to the extent that is closer to the original language; albeit, we acknowledge that some information and features carried by the local language might be lost when such verbal expressions are translated into English.

Thematic content analysis was performed following Graneheim & Lundman (2004) [35]. Data were explored manually using open codes. Transcripts were progressively coded by the first author [DO] to identify emerging concepts. This involved thorough reading of all client responses in every transcribed page, while considering the field-notes. A code-book was created based on the transcripts from the first two focus groups, and updated subsequently if new concepts appeared. The codes were subsequently reviewed again by DO purposely to identify and refine the relationship among the codes and subsequently categorize them broadly based on conventional literature to reflect important emerging ethical concepts related to ethics of screening populations for disease. Participant experiences and views on screening for both Syphilis (compulsory) and Cancer of the cervix (optional) were respectively extracted from the text based on the derived codes, condensed and subsequently sorted out by relevant content areas based on the overt and latent emphases as well as their unique experiences and expressions. Conceptual categories closely related to ethics of screening populations for disease were developed from the resulting themes, and illustrated by selected quotes.

## Results

The resulting themes were organised under the following content areas, to reflect emerging ethical issues in compulsory screening for syphilis and voluntary one for cancer of the cervix: lay knowledge and awareness of disease and risks; information-giving at pre-screening and disclosure of results; informed consent for the screening tests; time burden; potential harms during the screening process; acceptance/approval of screening approach.

### Participant experiences with and perspectives about screening for syphilis as a mandatory exercise

#### Knowledge and awareness of syphilis, sources of risks and its prevention

Majority of the women had satisfactory level of lay information about Syphilis. Their main sources of knowledge included the pre-clinical group sessions conducted by medical staff and awareness campaign events such as poster distribution. They understood that syphilis is an insidious sexually transmitted disease, sometimes associated with genital wounds, and it is treatable if action is taken promptly, albeit there were notable misconceptions. The following statements convey true facts

‘It is a sexually transmitted infection which is painful’

‘You can come to the hospital when pregnant or sick to be treated for it so it does not spread to the whole body’

However, it was notable that there were misconceptions indicating potential for confounded information:

‘It is transmitted by sharing pants with an infected person or passing urine in the same place where an infected person had urinated’;

‘If it overstays in your body, it can lead to AIDS’

Some participants also indicated that they don’t know about the disease despite having heard about it.

‘I don’t know about it, but I have heard about it’

Participants indicated that syphilis is a dangerous disease being associated with severe adverse natal, obstetric, medical and social sequelae. Such adversities include barrenness, loss of fetus, strained relationship between spouses and even death.

‘It is a dangerous disease, if you contract it you cannot get pregnant’;

‘If a close relation has this disease, I will feel bad because it is not good in life … I will advise her to go to the doctor quickly before the disease worsens to a condition she cannot walk…it can kill her so she should go to the doctor quickly!’

They also perceived that Syphilis can be latent in the body therefore, one should know her status by going for medical checkup and seek treatment if found to be positive or has recognized a symptom. Alternatively, if treatment fails, one could use herbs.

‘Before it is detected, you may not realize you have it; but if you are aware, you can go to the hospital and be treated’;

‘It affects the womb. … So it should be detected early and treated’

Consequently, participants considered syphilis to be worth paying attention to by individuals and entire communities given it is infectious and is associated with serious adverse health and social effects.

Based on their lay knowledge of the diseases, personal experiences and fear of adverse health effects, participants indicated that screening is beneficial to detect diseases early hence one is able to plan for appropriate treatment and prevention strategies. They further recognized early detection can be realized through medical check-up and recognition of symptoms based on statements below.

‘Before it [Syphilis] is detected, you may not realize you have it; but if you are aware, you can go to the hospital and be treated’

‘You can go to the hospital to be screened then start on medication if found to be positive’ … if it doesn’t work, use herbs’.

If you have a genital wound, go to hospital for screening’

Likewise they considered that ‘*taking control of one’s behavior to protect self after screening; creating awareness among the community members’* about the illnesses; use of condoms and seeking medical treatment promptly were pragmatic responsibilities for both the individual and whole community in managing syphilis.

### Perceived advantages and potential harms of Screening

When asked about what they knew about screening for syphilis, the participants stated that knowing one’s status provides a reasonable basis for making sexual and reproductive health decisions.

‘I know the disease exists and screening helps me in making [a] decision before engaging in sex with a man’

Furthermore, knowing that others have benefited from screening may motivate uptake.

It helps people go for treatment especially when the other people who are reluctant to go will go after they know they are not alone in this’.

On the other hand, reporting positive disease status was deemed a frightening experience. It heightened a sense of fatalism, dejection, worries, agitation and apprehension, hence need for supportive post-screening counseling.

‘If you are tested and not counseled, you can be shocked’

Although there was general confidence about securing the specimen through appropriate labeling, a few were still concerned about potential for getting ‘*wrong results’* in case of mix-up in specimen labeling.

*‘I am satisfied with the information given after the results because they know your results … when they take your specimen, they label them, hence you are sure the results you get are yours’*.

### Information disclosure at pre-screening and post-test period

#### Pre-screening information disclosure

The discussions revealed a split in opinion and experience about disclosure and information-giving approaches by the health staff at the clinic. Some of the participants indicated that the health staff at the clinic informed them about Syphilis and a range of tests to be done on them. Others also stated that their understanding about the disease had improved after undergoing the screening procedure. However, some were either distracted from attention or were not informed at all about the procedures.

Pre-screening information disclosure and counselling was considered essential for decision-making and minimizing risk of severe emotional consequences that may be associated with the process.

‘If am tested and am not counseled, maybe I can even refuse because it will make me think that these people are assuming I have Syphilis and if am counseled, I can just agree to be tested’

‘I have a better understanding because they test your status and teach you about caring [of your] private parts’.

A few participants expressed need for prior information about screening to facilitate involvement of spouses/sexual partners:

‘We were not aware of it. It is better if they inform us early so that we can involve our partners instead of being tested alone and my partner is not there’.

Most participants considered pre-screening disclosure helpful but preferred sessions to be interactive, private and confidential. They expected the care-giver to provide sufficient, explicit, simple and clear information in a manner easy to understand.

‘… [T] here is little room for interaction and some talk too fast and clients too many to allow time for discussions … so they want you to give other people room’

However, a few participants were indifferent and others more critical or guarded about the benefit of the disclosures. They were particularly uncomfortable if information about procedures was unclear or when they perceived that care-givers were impersonal and lacked opportunity to engage with them.

‘They didn’t discuss it with us; they just checked the status, so we are blank’

‘It depends on how you find the doctor, how she/he talks to you’.

### Disclosing/sharing test results and post-test information giving

The information given is verbal, but the results are written in a book which the clients carry home (usually the outpatient card). Written results were considered impersonal if not verbally reinforced. There was a sense of fear or anxiety about the outcome of the tests. Even so, a majority appreciated the fact that results are given to each individual at a time and in private. They were also content with the level of confidentiality accorded since results are given *‘secretly’* (in private and in confidence).

‘*I was happy and satisfied because we talked the two of us and no one knew what we had talked about* ’

Being aware that respective specimen bottles are labelled correctly enhances their confidence about the correctness of the results given, hence their trust in the process.

*‘I am satisfied with the information given after the results because they know your results … when they take your specimen, they label them, hence you are sure the results you get are yours’*.

But some were uneasy because of a perceived potential for the results to be mixed up, in case there was a problem with labeling of the samples. Consequently they preferred that *‘they [staff] should not rush [in case they] give wrong results’*.

Given the prospects for psycho-emotional stress from the test procedures, there was expectation for psychological support after the disclosure of results to help them cope.

‘If results are positive, you should be given guidance and counseling politely and not harshly or by throwing words’

‘It depends on how you find the doctor, how she/he talks to you’.

Nearly all participants underscored necessity to conduct disclosure privately ‘*with respect, dignity and politely*’. They were concerned that sometimes, the staff communicated the test results inappropriately. A friendlier staff demeanor particularly a female was more preferable to many.

### Disclosure of test-outcomes to relatives

In discussing whether they would disclose the test results to their spouses or close relatives, it emerged that confidentiality, discretion, intimacy and trust were necessary aspects in disclosing Syphilis test results, specifically by the individual patient.

‘It’s better if its I who breaks the news to my husband and not anyone else, because in the hospital we are told the results are confidential’

‘If it’s your husband, it’s okay … because you will be able to plan your life and know how to stay, because you stay together and it’s important that he knows’

The picture was made complicated by the feelings related to the tests. Participants expressed acceptance and appreciation, a sense of relief, expectation of effective treatment, worries, fatalism, dejection and agitation. They felt that the clinical area should also guarantee privacy of clients to assure their confidence:

‘I had thought about it … I knew if I had it then I was HIV positive’

### Informed consent

Opinions were varied about submitting to screening for Syphilis during antenatal visits and whether the information should be written or just verbal. Written consent was considered necessary to authenticate decisions, clinic visits and specify tests to significant others. However, simultaneous multiple clinical tests confounded the context for informed consent specific for Syphilis. While a number of respondents were aware antenatal tests for Syphilis was mandatory, few were ready for them while some felt providers ‘*pushed’* them into it and others were ambivalent as revealed by the statements below.

‘It is a disease that must be screened for during pregnancy, so if you are free you just come, you don’t wait to be told’;

‘I had made a decision to be tested before I got pregnant, but at the clinic I was told it is a must’.

‘We were told it is a rule to undergo the test … even if you did not come for the screening’

Nevertheless, there was broad consensus that ‘informed consent’ process if conducted in privacy, accords the clients an opportunity to clarify their positions before opting for the test procedures.

*‘I have two minds. One, it is good since the disease is treatable. Two, I feel stressed, because I will be asking myself – where did I get it from’*.

While some of the clients came purposely for screening, others felt they didn’t have a choice since it is compulsory for everyone who attends antenatal clinic. Participants indicated need to clearly understand the test processes and prospects, without being coerced or handled in a manner that may impair personal judgment, given that decision-making is a complex affair. While some participants acknowledged improved understanding of the disease, others felt that they were not prepared initially before coming to the clinic and only obeyed what they were told to do while others indicated they were threatened and others were concerned with the harsh or judgmental attitudes of the staff as revealed by the following statements.

‘It is a disease that must be screened for during pregnancy, so if you are free you just come, you don’t wait to be told’;

‘We were told it is a rule to undergo the test … even if you did not come for the screening’;

‘I had made a decision to be tested before I got pregnant, but at the clinic I was told it is a must’

‘A fine will be imposed if [they] don’t concentrate and later [can’t] seek for more consultations’.

### Time and cost burden

Most of the participants felt the waiting time before getting results was too long, sometimes running to a whole day or several weeks, for cervical cancer screening. This further heightened their anxieties as well as discouraging them from coming back to the clinic for follow up. Majority of women coming for screening of cancer of the cervix had travelled from far. Some reported the process was complicated and took long to complete. Lack of money to pay for some of the services and/or travelling (cost factor) further complicated their choices for the screening services.

### Experiences during test procedures

Overall, participants highlighted diverse experiences and feelings. On a positive note, some of them felt accepted and appreciated during the process, conferring a sense of relief about results and expectation to get effective treatment. Participants who initiated screening felt more confident the services were helpful. Nevertheless, others experienced worries, despondence and sadness. Adverse perceptions or experiences during screening were reportedly the reasons some women opted instead to seek alternative care from traditional midwives who do not perform screening. It emerged that those who ‘*don’t know their status’* and don’t attend clinic were likely to influence others against screening.

### Necessity and acceptability of repeat tests and follow-up visits

There were varied perceptions about follow-up or repeat examinations. While some appreciated that it may be necessary, in case of missed detection at the initial visit, others were not convinced of its value.

‘I can just go back if required because I don’t know why and just in case I have the disease but it was not detected at initial screening, so its good to know what is happening’

Some were unhappy with it because of the potential to feel more anxious and worried, in addition to the long waiting time.

I can just go but asking myself “but I had already undergone the test; why are they telling me to repeat it?” May be I got it from somewhere!’

‘It would be difficult if [I] am going to spend the same time waiting for results’

Others detested the whole idea because of perceived incompetence of the process, previous adverse experience, negative attitude and potential for raised anxiety.

“I will not accept [repeat tests] because she tested me the first time. Is it that she didn’t get it? I am not at ease since it’s already tested …’

‘I can’t be happy because the initial results were bad. So I can’t be sure of my life’

‘I can’t be happy because the initial results were bad. So I can’t be sure of my life’

‘I cannot come back … I don’t know doing what’

Some of the participants’ key concerns with screening were: long waiting-time, the potential to test positive (fear of outcomes), lack of coping skills or psychosocial support for those who test positive due to insufficient community awareness, ‘*some are not allowed by their husbands’* to attend clinics and potential for stigma due to disease being associated with sexuality. Some showed apprehension with potential for loss of confidentiality, in a case where the ‘*staff attending to me is familiar but I would not want my status revealed*’. These may contribute to reluctance to comply with follow-up visits among those who test positive.

### Client perspectives and experiences with voluntary screening for cancer of the cervix

#### Knowledge and awareness of Cancer of the cervix, sources of risks and its prevention

Clients included those who were referred by clinical staff, self-referred and others were coming for repeat testing. Cancer of the cervix was considered as a serious and *‘disturbing’* condition affecting reproductive organs.

‘It affects the inner part you can’t know, except the doctor who tests it know what it is’

‘It sucks blood and it causes wound to the affected area’

They largely associated it with ‘*too much sex*’ as well as to familial inheritance, poor genital hygiene, smoking cigarettes and abortion.

It was related to unusual ‘menstrual-like’ symptoms:

‘you experience abdominal pains, blood and water comes out; like me an old lady like me I had stopped giving birth long time ago, I stopped menstruating many years back, so that it is disturbing me like the way they’ve screened it, what will they do?’

Their motivation for learning more about the disease included personal experiences, individual needs and perception of the disease motivated them to them to learn more about the disease.

‘… like I had not come for the screening [of cancer of the cervix] and I didn’t know the disease could be detected, so it is advisable to go for screening even before seeing the signs’

### Disclosure of information at pre-screening and post-test

#### Pre-test information disclosure

Disclosure was necessary to demystify disease causes:

‘Me, I think that I have better understanding than before, because I used to know that cancer is caused by cigarettes, I learnt that the disease is not caused by cigarettes alone it can be inherited from family background and also if you see that it’s in your family lineage its better you go for screening in time so as to know if you don’t have it or if you have it know how you can be helped in time’

Majority appreciated that the sessions were private and confidential and that the information provided was sufficient. Adequate information enabled them to seek prompt medical intervention, spiritual and emotional support.

However, others were concerned with lack of information-giving and being asked questions which were considered as intrusive, irrelevant or too complex for them to understand:

‘*I was asked about the last time I gave birth, but I had forgotten since it was a long time ago*’.

Others indicated that they already knew what they wanted irrespective of the information received at the clinic.

The participants were concerned with effectiveness of the test procedures, the possibility that existing symptoms similar to that of Cancer of the cervix might complicate the picture and disclosure and sufficiency of the information disclosed before and after the test results. Additionally, they raised concern with long wait for results and difficulty in coping with positive test results.

### Post-test information disclosure

Most of the participants felt that test results should be released promptly, to minimize anxiety, and in a language that is easy to understand. Written results were considered impersonal if not verbally reinforced, especially since some do not know how to read.

‘you should be told about your results immediately instead of waiting, since after the test, when not told you don’t know the results and your heart is not settled’

Some of those who obtained negative test results felt that they were not given additional relevant information about the disease and what to do next (possibly feeling ignored), hence raising concern about the possibility of false negative test results. Positive test results presented the greatest challenge to handle, because cancer inherently induces fear, anxiety and loss of hope both to self and the family in addition to the possibility that the disease is already advanced. This may be further complicated by inadequate system for care and support:

‘If you know your status, you won’t live long. The shock will kill you. So you are better off not knowing’

‘When the nurse told me to lie down to be screened, she said that my case is hard and I had to wait for the other doctor whom she called to check on me. I believe in [G]od; so according to [H]is will so be it, am waiting for the other doctor’

However, some were more confident and optimistic about their post-test care and coping process:

‘Mmh, now, if they get us with the disease its good because now I can be given medication, like now I have been told to come on Tuesday to the clinic’.

‘… before getting inside you are told everything, so when you come out of that place its you to accept or deny, but me what I see its good, you accept the result, come to terms with the situation and leave things to God and life will not be difficult, but if you live in denial you will shorten your life span and starts to regret that I wish I knew , I would not have gone for this test, and being told about this disease’

### Disclosure of known status to relatives

Disclosing the test results to close relations was generally considered positively because of the potential social support this would elicit, but this may vary depending on the bestowed or positional responsibilities in the family. It was more preferable for the client herself to divulge the information:

‘*I will prefer to be the one to tell them since I know how they are. Some may be shocked and distressed*’.

### Informed consent

Participants elicited satisfaction with disclosure of information and trust during the procedures as being important for decision to submit to screening for cancer of the cervix. However, confidentiality and ‘trust’ in the caregivers (particularly those familiar to them) not to divulge personal details was considered crucial in accepting procedure. To enable them make meaningful decisions the information disclosed should be relevant, in a language that is easy to understand and individualized to the needs of the clients, sufficient in content with *‘thorough explanations’* to meet the individual’s needs (depending on health status and the anticipated test-outcomes), interactive and provided in a welcoming environment. It was noted that clients may come with fixed mindset while others may find it difficult to concentrate on the information provided about the disease and the tests.

There was a sense of ‘I am doing this because ‘the doctor had said’, but I don’t have a choice’. A few participants felt they were ‘*pushed*’ by the doctor to take the tests. Alternatively, they’d submit because of being already too frail and desperate for help with their symptoms. Hence, it was difficult to know whether submitting to a recommended test was truly voluntary.

### Follow up and repeat tests for surveillance

Some participants considered repeat tests necessary to identify the disease *‘you are suffering from’* is known but, for some this would be unacceptable:

‘I will not accept [repeat tests] because she tested me the first time. Is it that she didn’t get it? I am not at ease since it’s already tested …’

Issues related to fear of the disease, ignorance, time burden, cost of travel and staff attitudes were elicited as potential factors that may compliance with follow up schedules.

### Pain and discomfort

Participants may feel discomfort and pain during the pelvic exam. A few may simultaneously feel uncomfortable if the males performed the pelvic examination particularly those who felt they had no symptoms because of embarrassment to undress before a *‘stranger’*.

*‘At first I felt embarrassed when the [male] doctor told me to undress and climb up the couch [to be done vaginal exam]; but the doctor talked well and urged me to be open so as to be helped’*.

However, they were reassured by the presence of a female assistant. Another cause of discomfort associated with emotional stress was largely due to anxiety during the long-interval before results were released.

‘… you should be told about your results immediately instead of waiting, since after the test, when not told you don’t know the results and your heart is not settled’

## Discussion

Understanding ethical perspectives of public health interventions is necessary to improve decision-making and address the diverse aspects which may create or exacerbate ethical problems [14,36]. The study indicates that whereas screening may be perceived by some as helpful, ethical complexities exist in relation to the impact of both voluntary and compulsory approaches. It is necessary for the national programs to restructure and/or further enhance the screening process to mitigate potential ethical problems as well as improve service uptake.

### Pre-screening information disclosure

Pre-screening information disclosure is critical to notify clients of a mandatory or potentially coercive public health intervention, its rationale and procedures involved and options [37]. This is necessary to complement client’s autonomy, gain their trust and enjoin them in decision-making to protect their interest [37]. In this study, screening process in both approaches involved registration at the initial encounter, pre-screening health talk; conducting medical and/or laboratory tests; communicating results and invitation to follow-up visits. The implication of this is that guidelines to mitigate the ethical challenges can be operationalized at each stage of the screening process. For example the disclosure process can be enhanced by adopting simple interactive discourse based on appropriate checklists or talk-aids, during the process to improve respect for participant autonomy and value preferences [38]. Effective messaging would help in illuminating lay understanding, clarifying notions and shaping decision intentions for on-going participation in the screening programs [39].

### Decision-making and participation in screening

Voluntary choice to participate in screening remains a subtle issue given the nuances associated with providing health services within contexts of diverse cultures, inherent inequities and systemic inadequacies [27]. Additionally, interposing gender preferences and relationships, communication challenges, time burden, nature of tests required and individual idiosyncrasies complicate decision-making for participants in compulsory screening. Such challenges can adversely affect choices during screening services [40,41].

It was observed that decision to consent for compulsory screening was influenced by multiple factors including compulsoriness of the procedure, individuals’ lay knowledge; social biases; sense of personal moral obligations; pre-screening education talks; pregnancy status; existing parallel tests; perceived health status and expectations to benefit from disease identification. While some women were ready to take the tests and easily approved the exercise, others were not and felt the process was coercive. Literature on screening shows that objective communication strategies are useful in augmenting client’s on-going participation in screening can [42,43] These may include including use of guided communication aids to improve understanding of target information [42,43] encourage informed choices. Additionally, privacy and confidentiality should always be guaranteed [44].

The desire for women undergoing compulsory screening (for Syphilis) to involve their spouses in the process either by prior information or providing written documentation was aimed to improve decision-making, minimize forms of prejudice, and enhance disclosure of results. This indicates the ‘corporate’ aspect of consenting for compulsory screening for women, often involving preference from key family members. It also reinforces the importance of couple participation in screening process. The goal is to clarify concerns and promote peer decision support in risk comprehension among participants [45]. Hence, understanding local value norms and inculcating them into the communication packages can augment compulsory screen procedures. Croyle and Lerman’s [46] observed that clients’ decision to seek or accept genetic screen tests and whether they would recommend it to their kinfolk was informed by their perception of risk, given their experiential or lay knowledge of the target diseases.

### Paternalism versus voluntarism

One of the likely sources of ethical tension in implementing compulsory screening programs is the balance between respect for personal choices and preferences to opt out of the tests vis-à-vis the public health objective to control syphilis in the population. On the other hand, under voluntary screening process, a health provider may be confronted with a dilemma in responding to an ambivalent client who cedes her decision-making to the care-giver, ‘trusting’ that a medical staff, would make ‘*expert’* decision in their best interest (reasonable standards criteria). In both cases, the national guidelines should be clear on how to reasonably proceed. These tensions demonstrate the inherent complexities encountered in bid to resolve competing demands between fulfilling client preferences (personal dignity) and professional obligations to minimizing public health risks (paternalism) [38,40,47]. Frequently, this position is reinforced by the power balance in favour of the service provider [48] as well as the clients’ perceived vulnerability or apparent helplessness towards diseases viewed as severe, painful, disturbing or dangerous. Hence, decision-makers and service providers need to constantly review national screening program guidelines and to undergo refresher training to progressively learn emerging issues.

### Confidentiality, privacy and disease notification

Potential conflict exist between regulatory requirement for mandatory disease diagnosis, treatment, notification and sufficient follow up/surveillance of Syphilis sero-positive cases and their partners [7,31,36,49] versus the concern for confidentiality, client autonomy and potential prejudice from partners. Primarily, the screening guidelines should clarify more clearly how staff should conduct the process and manage the potential dilemmas, bearing in mind the legal provisions. For example, an algorithm may be provided for quick reference by service providers. Also, policy-makers at the global or national policy levels should apply more stringent considerations for balancing benefit and harms when designing the required guidelines. The goal is to minimize potential infringement and harm in case individuals plus their families are identified as affected or being at an increased risk [12]. On the other hand, they should be simple to follow.

### Pain, psycho-emotional and social harms associated with screening

The potential to feel pain, a measure of physical or emotional discomfort during or after procedures, and fear of being stigmatized due to screening procedures or test status requires a delicate balance between the health needs within the prevailing contexts and the goals of the screening exercise. The ethical requirement is to have and comply with guidelines, which should be comprehensive and displayed for ease of reference [6,7,22,50] during pre- and post-screening counseling. Additionally, providing relevant training and regular updates for the staff involved is essential. Additionally, all clients attending respective screening clinkics should be furnished with well-designed, comprehensive yet simple to follow education aids containing information about the target disease(s) to enhance understanding and allay anxieties. Receptionists or front desk-staff who are often the first (and last) contacts with the clients should be appropriately trained and skilled in communication process.

### Time burden

Time burden (as a hidden cost) constitutes the period that clients have to spend going through the entire process of screening until disclosure of results [51]. Prolonged waiting time may potentiate uncertainty or exacerbate clients’ existing apprehensions especially when no adequate preparation or information is provided. Also, time could be experienced as problematic for clients, when limited or lacking, forcing hurried services and possibly reinforcing their perception of care as impersonal and insecure. This poses a real barrier to service uptake. Prolonged travel and waiting time as well as related costs were barriers to uptake of screening services as they augment the time burden incurred by clients.

### Limitations of the study

The study did not consider the perspectives of the providers about compulsory and voluntary screening. Hence it was not possible to know the challenges and how they resolve them.

The participants interviewed at the clinic were likely a self-selected group compared to those who don’t attend services at the health facility, hence need for a field survey to clarify these aspects.

## Conclusions

The context for mounting screening as a public health intervention and attendant ethical issues may be more complex than hitherto perceived. The study revealed that individual context is critical to decision-making. This indicates that, at the global and operational level, interpreting emerging ethics issues in screening requires more nuanced considerations of individuals’ mundane experiences that are innately intertwined with their traditional and contemporary lore.

It is apparent that in considering mounting screening for Syphilis (mandatory screening) and cervical cancer (voluntary screening), the community interests and perspectives may be at variance with that of the national health regulatory and policy frameworks. This is bound to influence both operational and demand dimensions of service delivery because of challenges to comply with prescribed guidelines on one hand and conformity to or cooperation with the social norms on the other. Besides, perceived barriers may cause clients to lose opportunities to clarify their concerns at the service interface, perhaps for fear of retribution or communication lapse whereas the staff may also fail to elicit and mitigate adverse personal or group experiences during such interactions. Hence, tensions between voluntarism and paternalism in screening may be reinforced.

Lack of clear messaging may further influence adversely the demand for screening services by the individuals at risk or the community as a collective agent. However, training on appropriate health education techniques is more likely to improve intended outputs of screening process. However, attention should also focus on the unapparent populations which may potentially influence social behavior.

The current study has also revealed that real harms exist during screening, whether mandatory or voluntary. However, effectiveness of the screening approaches vis-à-vis the harms still require further evaluation across diseases in different service delivery and geographical contexts.

## Competing interests

The authors declare they have no conflict of interests.

## Authors’ contributions

DSOA designed and executed the study, processed and analyzed the data and drafted the manuscript; NM participated in designing the original study concept and supervised the study. Both authors read and approved the final manuscript.

## Pre-publication history

The pre-publication history for this paper can be accessed here:

http://www.biomedcentral.com/1472-6939/15/27/prepub

## Declaration

I declare that this manuscript has never been submitted to any other journal for consideration for publication. The authors are responsible for the entire contents of the manuscript.
